# Primary hyperoxaluria diagnosed after kidney transplantation failure: lesson from 3 case reports and literature review

**DOI:** 10.1186/s12882-019-1402-2

**Published:** 2019-06-18

**Authors:** Ruiming Cai, Minzhuang Lin, Zhiyong Chen, Yongtong Lai, Xianen Huang, Guozhi Zhao, Xuekun Guo, Zhongtang Xiong, Juan Chen, Hui Chen, Qingping Jiang, Shaoyan Liu, Yuexin Yang, Weixiang Liang, Minhui Zou, Tao Liu, Wenfang Chen, Hongzhou Liu, Juan Peng

**Affiliations:** 1 Department of Organ Transplantation, The Third Affiliated Hospital of Guangzhou Medical University, Guangzhou, 510150 China; 2 Department of Pathology, The Third Affiliated Hospital of Guangzhou Medical University, 63 Duobao Road, Guangzhou, 510150 People’s Republic of China; 3 Department of Ultrasound, The Third Affiliated Hospital of Guangzhou Medical University, Guangzhou, 510150 China; 4 Department of Pathology, The First Affiliated Hospital of Sun Yat-sen University, Guangzhou, 510080 China; 5 Department of Clinical Laboratory, Guangzhou Kingmed Center for Clinical Laboratory Co., Ltd, Guangzhou, 510330 China

**Keywords:** Primary hyperoxaluria, Kidney transplantation failure, Calcium oxalate crystals, Case report

## Abstract

**Background:**

Primary hyperoxaluria (PH) is a rare inborn disorder of the metabolism of glyoxylate, which causes the hallmark production oxalate and forms insoluble calcium oxalate crystals that accumulate in the kidney and other organs. Since the manifestation of PH varies from recurrent nephrolithiasis, nephrocalcinosis, and end-stage renal disease with age at onset of symptoms ranging from infancy to the sixth decade, the disease remains undiagnosed until after kidney transplantation in some cases.

**Case presentation:**

Herein, we report 3 cases of PH diagnosed after kidney transplantation failure, providing the comprehensive clinical course, the ultrasonic image of renal graft and pathologic image of the biopsy, highlighting the relevance of biopsy findings and the results of molecular genetic testing. We also focus on the treatment and the unfavorable outcome of the patients. Meanwhile, we review the literature and show the additional 10 reported cases of PH diagnosed after kidney transplantation. Additionally, we discuss the progressive molecular understanding of the mechanisms involved in PH and molecular therapy.

**Conclusions:**

Overall, the necessity of preoperative screening of PH in all patients even with a minor history of nephrolithiasis and the importance of proper treatment are the lessons we learn from the 3 cases, which prompt us to avoid tragedies.

**Electronic supplementary material:**

The online version of this article (10.1186/s12882-019-1402-2) contains supplementary material, which is available to authorized users.

## Background

Primary hyperoxalurias (PH) are a group of rare autosomal recessive diseases characterized by the overproduction of oxalate resulting from hereditary enzymatic defects in glyoxylate metabolism, which currently include 3 known types [1–3]. Of the primary hyperoxalurias, approximately 70% are PH type 1 (PH1), 10% are PH type 2 (PH2), 10% PH type 3 (PH3), and 10% do not have an identified genetic cause [4]. PH1, which is the most frequent and serious disorder due to enzyme deficit of alanine-glyoxylate aminotransferase (AGT) specific to hepatic peroxisome, is determined by mutations in the *AGXT* gene. PH2 and PH3 are respectively caused by a deficiency of glyoxylate reductase/hydroxypyruvate reductase (GR/HPR) encoded by *GRHPR* gene and 4-hydroxy-2-oxoglutarate aldolase (HOGA) encoded by *HOGA1* gene. Oxalate is excreted through the kidneys, where excessive oxalate precipitates in the form of calcium oxalate (CaOx) crystals, leading to recurrent nephrolithiasis, nephrocalcinosis, chronic kidney disease (CKD) and eventually end-stage renal disease (ESRD) [5]. However, in some cases, high diagnostic suspicion of PH is proposed after renal graft loss. Here, we present the main steps of 3 cases in the treating experiences of the disease and the management strategies that have been used to control the recurrence of PH at this time. We expect the report might bring more attention to other patients with the same situation.

## Case presentation

### Case 1

A 27-year-old male hypertensive patient was referred to our department on Nov 15, 2017, after right nephrectomy as well as left minimally invasive percutaneous nephrolithotomy (mini-PCNL). With a history of symptomatic kidney stones for more than 10 years and elevated SCr level for more than 3 years, accompanied with hypertension (peak 170/98 mmHg), the patient was scheduled to receive the renal transplantation.

**Fig. 1:**
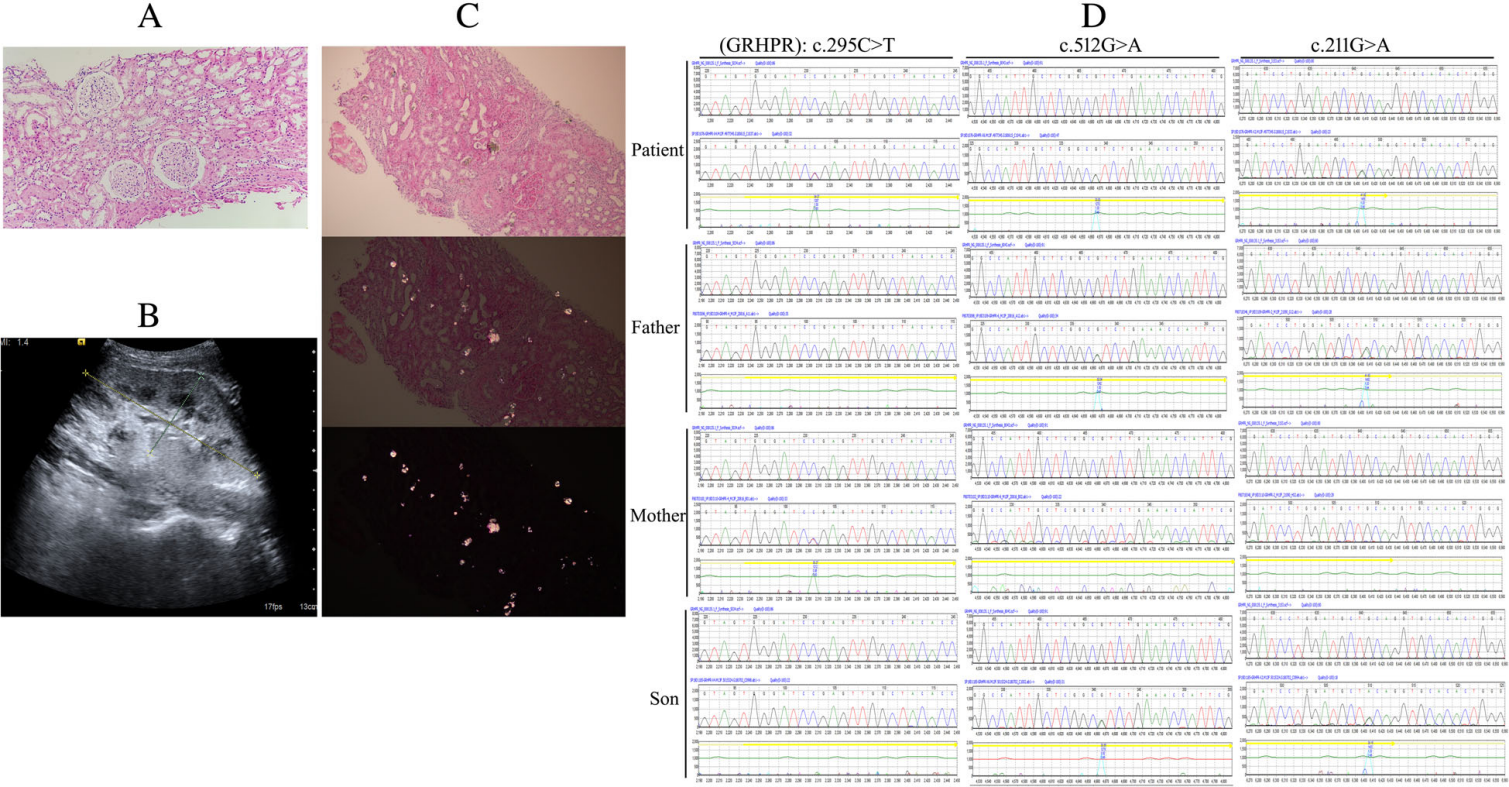
Clinical data of Case 1. **a** ZHIB of the renal allograft showed no crystals (200X HE). **b** USG of performing renal graft biopsy. The renal volume was increased obviously than normal. **c** Renal graft biopsy on post-op day 46 revealed acute T cell-mediated rejection (TCMR, 2015 Banff 1A) and extensive tubular damage, with the presence of the emerging deposition of CaOx crystals in the allograft (200X HE and polarized light). **d** Sequencing analysis of *GRHPR* gene mutations identified in the patient’s family. Three missense mutations in the *GRHPR* gene were found: first, a C to T transversion (c.295C > T) in exon 4 resulting in p.(Arg99*), nonsense PV; second, a G to A transversion (c.512G > A) in exon 6 resulting in p.(Arg171His), missense VUS; third, a G to A transversion (c.211G > A) in exon 2 resulting in p.(Ala71Thr), missense VUS

The patient was rehospitalized on Apr 28, 2018, with SCr 1487 umol/L and BUN 33.47 mmol/L (shown in Additional file 1: Figure S1). He underwent renal transplantation that night, with a deceased donor, which was performed with Zero-Hour Implantation biopsy (ZHIB, as part of the routine renal transplant procedure, shown in Fig. 1a, 200X HE). The patient received standard immunosuppression with mycophenolate mofetil (MMF), tacrolimus (Tac) and methylprednisolone (Methylpred) besides hemodialysis (HD), as well as hypertension treatment. Additionally, he received the follow-up assessments including routine blood tests, blood biochemical analysis and therapeutic drug monitoring regularly (Additional file 1: Figure S1). However, it was less likely to have improvements in the renal graft function.

Due to the delayed graft function (DGF, SCr 585 umol/L and BUN 30.07 mmol/L), the patient received percutaneous needle core biopsy of renal graft on post-operative (post-op) day 46, guided by ultrasonography (USG) (Fig. 1b). Renal graft biopsy revealed acute T cell-mediated rejection (TCMR, 2015 Banff 1A) and extensive tubular damage, with the presence of the emerging deposition of CaOx crystals in the allograft (Fig. 1c, 200X HE and polarized light). Compared with ZHIB, recurrence of CaOx nephropathy was confirmed in the allograft kidney. Molecular genetic testing of the patient was carried out to identify the pathogenic variants (PV) in *AGXT* and *GRHPR*, in whom a homozygous genotype for three missense mutations in the *GRHPR* gene was found: first, a C to T transversion (c.295C > T) in exon 4 resulting in p.(Arg99*), nonsense PV; second, a G to A transversion (c.512G > A) in exon 6 resulting in p.(Arg171His), missense variant of uncertain significance (VUS); third, a G to A transversion (c.211G > A) in exon 2 resulting in p.(Ala71Thr), missense VUS (shown in Table 1 and Fig. 1d). Based on the previous findings a diagnosis of PH2 was made.

**Table 1:** *GRHPR* gene Mutations identified in the patient’s family

	Age	*GRHPR* gene			kidney stones
		c.295C > T (p. Arg99Ter), nonsense PV	c.512G > A (p. Arg171His), missense VUS	c.211G > A (p. Ala71Thr), missense VUS	
the patient	27Y	+	+	+	+(symptomatic)
the patient’s father	52Y	–	+	+	–
the patient’s mother	50Y	+	–	–	+(asymptomatic)
the patient’s son	2Y6M	–	+	+	–
the patient’s wife	25Y	–	–	–	–

We further analyzed the DNA of the patient’s family. Sequencing analysis of the *GRHPR* gene showed that the PV (c.295C > T) was also found in the patient’s mother, who presented asymptomatic kidney stones. Unfortunately, the other two VUS were detected both in the patient’s father and son (shown in Table 1 and Fig. 1d).

The patient was treated with Pyridoxine (PN, Vitamin B6) and temporarily intensive HD besides the basic immunosuppression to suppress oxalate overproduction. Furthermore, for the failure of kidney-alone transplantation (KAT), we recommended the treatment scheme with the combined Liver-Kidney Transplantation (LKT) before the development of systemic oxalosis but was refused by the patient. The patient currently reentered to the maintenance HD in the clinic and looked forward to a chance of LKT in the future.

### Case 2

**Fig. 2:**
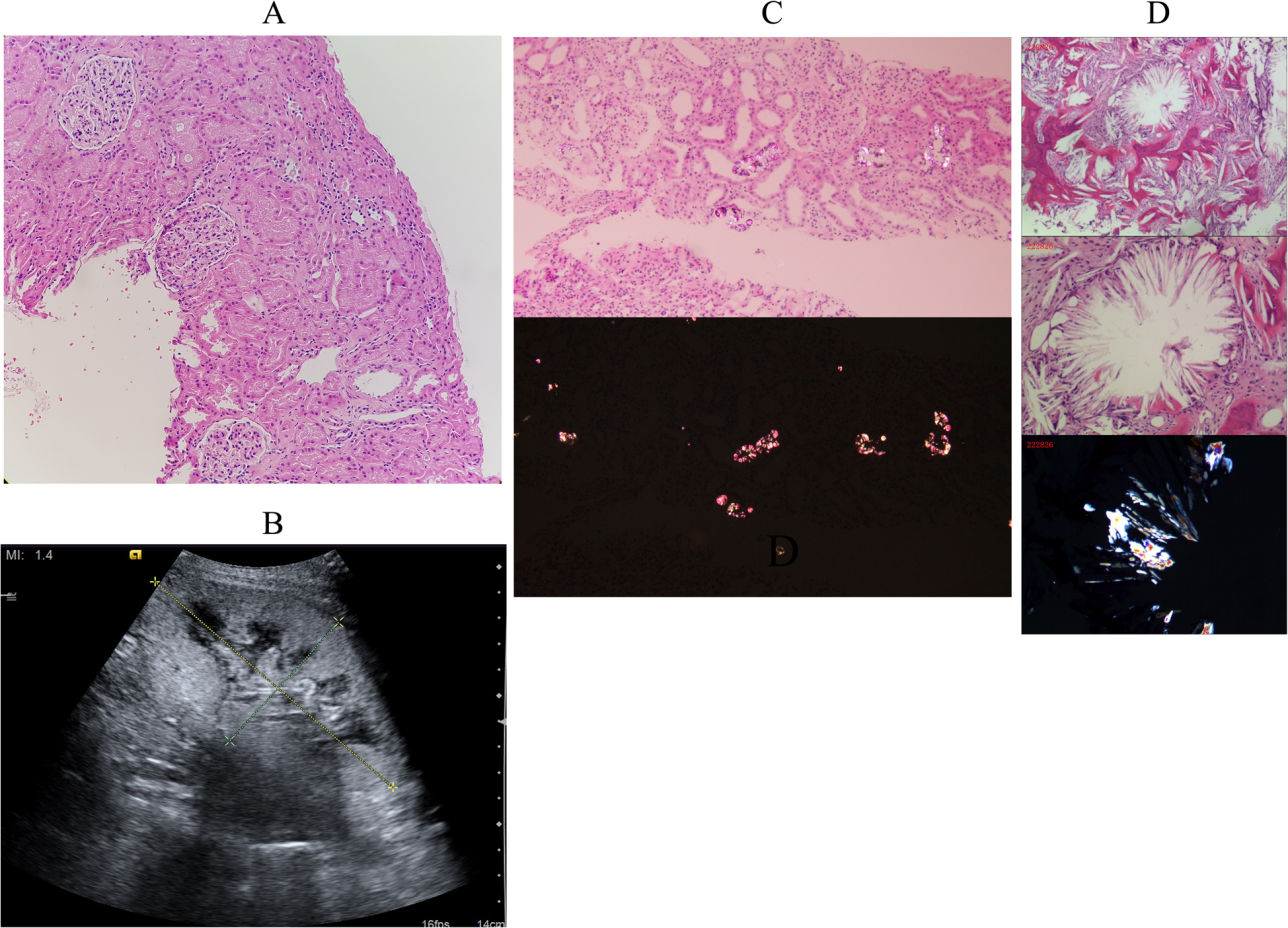
Clinical data of Case 2. **a** ZHIB of the renal allograft showed no crystals (200X HE). **b** USG of the renal allograft. The renal volume was increased obviously than normal. **c** Renal allograft biopsy on post-op day 38 displayed the deposition of diffuse CaOx crystals as well as acute TCMR (2015 Banff 2A, 200X HE and polarized light). **d** Bone marrow biopsy on post-op day 70 showed intertrabecular spaces occupied by abundant CaOx crystals (200X HE, 400X HE and polarized light)

A 26-year-old male non-hypertensive patient was hospitalized on Aug 11, 2016, with an 8-year history of the elevated SCr, which included a 7-year history of maintenance HD. The next day, attributed to the preoperative SCr 893 umol/L and BUN 27.32 mmol/L, shown in Additional file 2: Figure S2), he was transplanted with a deceased kidney donor performed with routine ZHIB (Fig. 2a, 200X HE). The patient received standard triple immunosuppression following transplantation as well as the follow-up assessments regularly. Similarly, even though we substituted Cyclosporine A (CsA) for Tac, renal graft gradually developed DGF (SCr 534 umol/L and BUN 30.06 mmol/L, Additional file 2: Figure S2), and USG-guided renal biopsy was employed on post-op day 38 (Fig. 2b). Deposition of diffuse CaOx crystals as well as acute TCMR (2015 Banff 2A) was detected in renal graft biopsy (Fig. 2c, 200X HE and polarized light), whereas there were no oxalate crystals in the ZHIB. Molecular genetic testing identified two mutations in the *AGXT* gene: first, exon 1: c.33dupC (p. Lys12fs), frameshift PV; second, an A to T transversion (c.215A > T) in exon 2 resulting in p. Asn72Ile, missense VUS. Meanwhile, it was noteworthy that one mutation in the *MUT* gene (Exon11: c.1897G > C (p. Val633Leu), missense VUS) was detected in the patient. Thus, the patient was diagnosed with PH1 and treated with PN (400 mg, iv, QD) and temporary intensive HD.

Owing to the anemia (hemoglobin level, HGB, 57–74 g/L; shown in Additional file 2: Figure S2), bone marrow biopsy was conducted on post-op day 70, which showed intertrabecular spaces occupied by abundant CaOx crystals (Fig. 2d, 200X HE, 400X HE and polarized light). Apparently, the patient was diagnosed as systemic oxalosis of PH1 with CaOx crystals involving both the allograft kidney and bone marrow, the latter is the most crippling site of CaOx deposition. He was treated with concentrated red blood cells (CRBC) transfusion and recombinant human erythropoietin (rhEPO) to correct the anemia. Moreover, as renal graft loss, he returned to the maintenance HD and received the optimal immunosuppression. Up till now, the patient was investigated for the overall decline in health following allograft failure.

### Case 3

A 34-year-old male hypertensive patient was admitted to hospital on Oct 16, 2015, for the first time because of the elevated SCr level for more than 12 months. Presented with SCr 1222 umol/L and BUN 24.33 mmol/L (Additional file 3: Figure S3) as well as hypertension peaked at 180/100 mmHg, the patient waited for the renal transplantation.

**Fig. 3:**
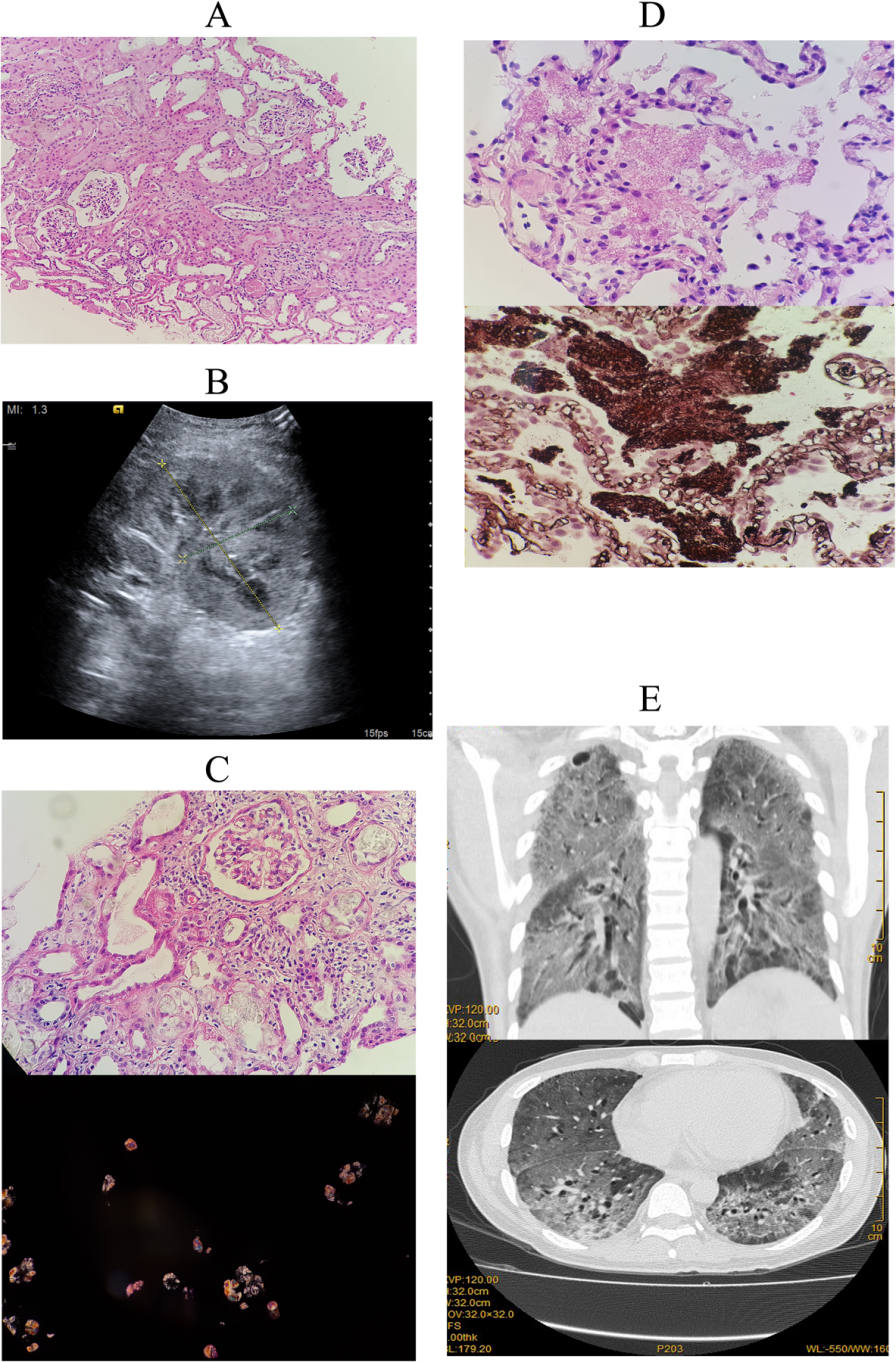
Clinical data of Case 3. **a** ZHIB of the renal allograft showed no crystals (200X HE). **b** USG of the renal allograft. The renal volume was increased obviously than normal. **c** Renal transplant biopsy on post-op day 75 documented acute TCMR (2015 Banff 2A) and extensive deposits of CaOx crystals in the interstitial tubule (200X HE and polarized light). **d** *Pneumocystis jirovecii* pneumonia (PJP) was confirmed in the patient on May 25, 2016 by fiberoptic bronchoscopic (FOB) biopsy, which showed *Pneumocystis* organisms (600X HE and Methylamine silver). **e** Chest CT examination demonstrated diffuse ground-glass opacities (GGO)

The patient was readmitted to hospital on Jan 5, 2016. He received a kidney allograft on the next day with a deceased donor performed with routine ZHIB (Fig. 3a), followed by post-op standard triple immunosuppression besides HD. Furthermore, the follow-up assessments were executed nearly once a day (Additional file 3: Figure S3). However, the course was unfavorable with DGF emerging. USG-guided renal graft biopsy (Fig. 3b) was performed on post-op day 75, which documented acute TCMR (2015 Banff 2A) and extensive deposits of CaOx crystals in the interstitial tubule (Fig. 3c, 200X HE and polarized light). In view of the fact that no oxalate crystals deposited in the ZHIB, as well as the history of kidney stones in the recipient, recurrence of CaOx nephropathy following kidney transplantation was confirmed, which led to the diagnosis of PH. However, the diagnosis of PH must depend on the genetic testing. Unfortunately, molecular genetic testing of the patient was not carried out to identify the PV in *AGXT*, *GRHPR* or *HOGA1* for some reasons.

Although the renal function of the patient was slowly repaired (SCr 260 umol/L; BUN 14 mmol/L), the treatment by HD was ineffective in treating excess oxalate besides the drug immunosuppression. Accompanied by the fever of 39.8 °C (Body temperature shown in Additional file 3: Figure S3) and intermittent cough, *Pneumocystis jirovecii* pneumonia (PJP) was confirmed in the patient on May 25 by fiberoptic bronchoscopic (FOB) biopsy (Fig. 3d, 600X HE and Methylamine silver) as well as subsequent Chest CT examination (Fig. 3e). The patient was initially treated with empirical anti-infection therapy (Micafungin) as well as γ-globulin. Meanwhile, standard triple immunosuppression was replaced with the pulse intravenous Methylpred. Even though the clinicians enhanced the anti-infection therapy with administration of trimethoprim-sulfamethoxazole (TMP/SMX) and voriconazole (VOR) orally, as well as meropenem (MEM) was used in the final stage, the patient died of severe pneumonia caused by *P. jirovecii* on Jun 28, 2016.

## Discussion and conclusions

Although the factors of DGF was various, it’s necessary to differentiate between DGF secondary to recurrence of PH or other reasons as soon as possible [6]. Given the manifestation of PH varies from recurrent nephrolithiasis and ESRD during childhood to occasional kidney stones in adulthood, especially to date systemic oxalosis has not been reported in PH3 [1–3], as well as the limitations of the routinely applied methods affect the diagnosis of PH, it still confused us to preoperative diagnosis of PH. Furthermore, the bleeding risk of percutaneous renal biopsy in the patient of ESRD also reduced the possibility to conclude the cause of disease. However, the diagnosis of PH in case 3 was not recognized until after renal transplant failure with fatal consequences, which led us to confirm the importance of selective screening before planning for kidney transplantation in the population with clinical and biochemical suspicion of PH. Molecular genetic testing was adopted for these people to identify the PV in *AGXT*, *GRHPR* and *HOGA1* gene, which were respectively the genetic determinants of PH1, 2 and 3. Despite we paid more attention to the similar situation, there were still overlooked or delayed affairs happened in our hospital such as case 1 and 2. Both patients had developed into the early post-transplant renal graft loss by DGF secondary to recurrence of PH.

Since Riksen et al. first reported the case of a Yugoslav renal transplant patient, who was confirmed the diagnosis of PH1 after a second renal transplant [7], we revealed an additional 12 reported cases of PH diagnosed after kidney transplantation by a comprehensive search of published English-language literature (Table 2) [8–15]. The early post-transplant renal graft loss, including a return to maintenance HD, re-transplantation, or patient death, had occurred in most cases. Considering the poor outcomes when crystalline nephropathy recurrence after transplantation [16], it is obviously optimal to recognize PH by molecular genetic testing as early as possible, in order to implement specific therapies and strategies aimed at lowering oxalate levels.

**Table 2:** Clinical Features of Reported cases of Primary hyperoxaluria diagnosed after kidney transplantation

Study	Age/Gender/Resident/Time/Type Diagnosed	Medical history	Clinical presentations	Biopsy of renal graft	Genetic testing/other proofs	Treatment	Outcomes
Riksen et al, 2002 [7]	51/M, Yugoslav. 2000. PH1	revealed an operation for urolithiasis at the age of 3, developed ESRD at the age of 40. His first kidney transplantation was performed in 1996, and the second in Sep 2000.	On post-op day 64 after the second renal transplant he was readmitted to the hospital for a rise of SCr level.	revealed numerous birefringent crystalline deposits in the proximal and distal tubules, arranged in a rosette-like array, consistent with CaOx crystals.	*Not detected.* *Biochemical urine analysis revealed elevated excretion rates of oxalate and glycolate, whereas no L-glycerate was present. POC was severely elevated.*	treated conservatively with a high fluid intake, avoidance of high oxalate foods, a thiazide diuretic, and a trial of PN.	Eight months after his renal transplantation, the creatinine clearance had stabilised at 21 ml/min and POC had decreased to 29 μmol/l.
Kim et al, 2005 [8]	43/F, Korean. 2004. PH1	shown renal dysfunction since 1991 without a history or symptoms of renal stones or other signs of systemic oxalosis. HD was initiated in Feb 2000. She received a renal transplant on Sep 12, 2002.	At post-op day 4 to 11, urine volume was abruptly reduced with the rebound of SCr level, eventually it appeared anuria.	Tubular lumens contained numerous rhomboid, polyhedral or cone-shaped crystals attached to the necrotic epithelial cells with calcium deposits.	*Not detected.* *Liver biopsy was performed at the end of Oct 2004 to measure AGT activity, which was 2.7 mmol/h/mg protein, and AGT immunoreactivity was negative.*	*Not stated*	Her renal function gradually worsened, leading her to receive HD.
Madiwale et al, 2008 [9]	25/M, Indian. *Not stated* PH	with ESRD due to bilateral, multiple nephrolithiasis. He received a renal graft from his mother	SCr rose from 1.1 mg/dL on post-op day 5 and was 2.6 mg/dL by the fourth week.	We reviewed slides of nephrectomy and graft biopsy and noted extensive deposits of birefringent CaOx crystals in the tubules, interstitium, vascular media and even the sclerosed glomeruli of nephrectomy	*Not detected.* *Skin biopsy showed oxalate crystals in dermal vessels.* *Autopsy revealed plentiful oxalate crystals in transplant kidney, myocardium, coronary artery, and in blood vessels of the spleen, pancreas and lungs.*	started with PN. Later, he developed oral candidiasis, necrotizing skin lesions on the lower limb, fever, abdominal pain, breathlessness and hemiparesis. He was treated with antibiotics and systemic anti-fungals and immunosuppression was reduced.	He developed hypotension and shock and died 10 weeks post-op.
Celik et al, 2010 [10]	38/M, Turk *Not stated* PH	with history of recurrent nephrolithiasis developed ESRD because of obstructive uropathy. After 2-year HD treatment, he received a deceased donor transplant.	The patient was re-operated on post-op day 13 because of slowly dropping SCr levels.	ZHIB showed only tubular vacualisation. The 13th post-op day allograft biospy showed intensive oxalate crystals deposition.	*In the fundus examination preretinal, intraretinal, and intravascular diffuse oxalate crystals were detected.* *In the multislice CT images, diffuse calcifications were observed in left anterior descending, left circumflex, and right coronary arteries.*	*Not stated*	In the 18th month, allograft biospy showed mild interstitial fibrosis and tubular atrophy with only a few crystals. The fundus examination showed the regressive nature of oxalate depositions.
Spasovski et al, 2010 [11]	48/F, Macedonia. 2007 PH1	presented with bilateral flank pain and signs of advanced chronic renal failure (CRF) in the beginning of 2006. In Jan 2007, she underwent a living unrelated paid transplantation.	From post-op 3 weeks to 4 months, she had a gradual increase in SCr levels from 299 to 423 μmol/l.	The second kidney graft biopsy showed ischemic tubular lesions and calcifications (oxalosis).	Heterozygote for 2 variants of the *AGTX* gene: -c.33_34InsC -c.508G > A	Since she was anuric, the monitoring of plasma oxalate was performed during a 2-month trial with increasing doses of PN up to 10 mg/kg/day.	The patient was put again on dialysis in July 2007. Nevertheless, she showed signs of systemic oxalosis.
Malakoutian et al, 2011 [12]	22/F, Iranian. *Not stated*. PH1	had the experience of two nephrolithiasis episodes 3 years earlier and had been under hemodialysis for 2 years later. She underwent kidney transplantation about 3 months before admission to our hospital.	After 2 months of transplantation, she presented with fever, malaise, vomiting, and a high SCr level.	There were large refractile acellular deposits in several of the tubular lumens resembling oxalate crystals making tubular destruction and injury.	Heterozygote for 1 variants of the *AGTX* gene: Exon 5: c.584 T > G: PV.	The patient then underwent hemodialysis via a jugular vein catheter despite conservative management.	The patient was discharged on maintenance hemodialysis.
Naderi et al, 2015 [13]	20/M, Iraqi. 2013. PH1	revealed an operation on the right kidney due to kidney stone at the age of 2.5 and several sessions of extracorporeal shock wave lithotripsy in the following years because of recurrent renal stones. At the age of 14, ESRD was established and he had been on regular HD. He received a renal transplant in Apr 2013.	On the 10th post-op day his SCr level started to rise and reached 1.8 mg/dL.	diffuse tubular deposition of CaOx crystals was seen.	*Not detected.* *The diagnosis was confirmed by low AGT enzyme activity on liver biopsy.*	treated with vigorous serum therapy and daily HD along with previous medications. However, the patient underwent graft nephrectomy on the 98th post-op day.	The patient is now under treatment with PN, potassium citrate and regular HD, looking forward to a chance of LKT in the future.
Rios et al, 2017 [14]	33/F, Colombian. 2011. PH1	presented ESRD associated with coral calculi in 2002, requiring bilateral nephrectomy and to start HD. She was first transplanted in 2004, and second transplanted in 2010.	One year after second transplantation, she presented a SCr elevation.	documented acute rejection of cellular Banff 1A and extensive deposits of oxalate in the interstitial tubule.	Heterozygote for 2 variants of the *AGTX* gene: -c.731 T > C (p. lle244Thr): PV. -c.307G > A (p. Gly103Arg): VUS, probably pathogenic.	treated with steroid boluses and conversion to Tac.	At her last follow-up at 6 years, her SCr was 4.8 mg/ dl; she is in pre-dialysis stage and being evaluated for LKT.
Rios et al, 2017 [14]	53/*Not stated,* Colombian. 2010. PH2	with a history of recurrent untreated nephrolithiasis who progressed to ESRD, which required to initiate HD in May 2008. A cadaveric donor kidney transplant was performed on July 9, 2010.	In Nov 2010, the patient presented renal function impairment.	showed Banff 2A acute cell rejection and extensive tubular damage, with presence of CaOx crystals and micro-calcifications.	Heterozygote for 2 variants of the *GRHPR* gene: -c.478G > A (p. Gly160Arg) -c.626C > T (p. Ser209Phe)	handled with pulses of Methylpred, ATG and conversion to Tac. Management with diet low in oxalate, PN, potassium citrate and HCT.	The renal function became progressively deteriorated until a terminal stage, with reentry to HD in May 2015.
Liu et al, 2018 [15]	33/M, Chinese. 2014. PH2	diagnosed with nephrolithiasis in Mar 2004. In Nov 2014, the patient developed dizziness and nausea with SCr 2500 mol/l. The patient was diagnosed with ESRD and underwent maintenance HD. He received an allogeneic renal transplant in Dec 2014.	On post-op day 15, serum creatine levels gradually increased to 291 μmol/l.	revealed borderline lesions of the renal graft and tubulitis accompanied by a small amount of crystal deposition within the renal tubules	Heterozygote for 1 variant of the *GRHPR* gene:	The renal graft was removed in Feb 2015. Renal pathology revealed interstitial injury due to crystal deposition within the renal tubules, with renal pelvis stones.	The patient received post-transplant HD and the date of the last follow-up visit was October 2015.
Current report case 1	27/M, Chinese. 2018. PH2	With symptomatic kidney stones for more than 10 years and elevated SCr level for more than 3 years, accompanied with hypertension, the patient was diagnosed with ESRD, obstructive nephropathy and kidney stones. After right nephrectomy as well as left mini-PCNL, he underwent renal transplantation on Apr 28, 2018.	Due to the delayed recovery of renal function on post-op day 46.	revealed acute TCMR (Banff 1A) and extensive tubular damage, with the presence the emerging deposition of CaOx crystals in the allograft.	Heterozygote for 3 variants of the *GRHPR* gene Exon4: c.295C > T (p. Arg99*): nonsense PV. Exon6: c.512G > A (p. Arg171His): missense VUS. Exon2: c.211G > A (p. Ala71Thr): missense VUS.	The patient was treated with PN and temporary intensive HD besides the basic immunosuppression.	The patient currently reentered to the maintenance HD in the clinic and looked forward to a chance of LKT in the future.
Current report case 2	26/M, Chinese. 2016. PH1.	With an 8-year history of the elevated SCr, which included a 7-year history of maintenance HD. On Aug 12, he was transplanted with a deceased kidney donor.	The allograft function did not obviously recovery on post-op day 38.	Deposition of diffuse CaOx crystal as well as acute TCMR (Banff 2A) were detected.	Heterozygote for 2 variants of the *AGXT* gene Exon1: c.33dupC (p. Lys12fs): frameshift PV. Exon2: c.215A > T (p. Asn72Ile): missense VUS. Heterozygote for 1 variants of the *MUT* gene Exon11: c.1897G > C (p. Val633Leu): missense VUS.	Treated with PN and temporary intensive HD. Due to the anemia, bone marrow biopsy was conducted on Oct 21, which showed intertrabecular spaces occupied by abundant CaOx crystals. He was treated with CRBC transfusion and rhEPO to correct the anemia.	He returned to the maintenance HD and received the optimal immunosuppression. Up till now, the patient was investigated for the overall decline in health.
Current report case 3	34/M, Chinese 2016. PH.	With the elevated SCr level for more than 12 months as well as hypertension. He received a kidney allograft on Jan 6, 2016.	The course was unfavorable with delayed recovery of renal function on post-op day 75.	documented acute TCMR (Banff 2A) and extensive deposits of CaOx crystals in the interstitial tubule.	*Not detected.* *In view of the fact that no oxalate crystals deposited in the ZHIB, as well as the history of kidney stones in the recipient, recurrence of CaOx nephropathy following kidney transplantation was confirmed, which led to the diagnosis of PH.*	Treatment by HD besides the drug immunosuppression. PJP was confirmed on May 25 by FOB biopsy. The patient was initially treated with Micafungin and γ-globulin. And immunosuppression was replaced with the pulse intravenous Methylpred.	Even though the clinicians enhanced the anti-infection therapy with administration of TMP/SMX and VOR as well as MEM was used in the final stage, he died on Jun 28, 2016.

*ESRD* end-stage renal disease, *CaOx* calcium oxalate, *post-op* post-operative, *HD* Haemodialysis, *POC* Plasma oxalate concentration, *PN* pyridoxine, *Tac* tacrolimus, *MMF* mycophenolate mofetil, *Methylpred* methylprednisolone, *CsA* Cyclosporine A, *ATG* thymoglobulin, *HCT* hydrochlorothiazide, *PV* pathogenic variant, *VUS* variant of uncertain significance, *LKT* combined liver-kidney transplant, *CRBC* concentrated red blood cells, *rhEPO* recombinant human erythropoietin, *ZHIB* Zero-Hour Implantation biopsy, *PJP Pneumocystis jirovecii* pneumonia, *FOB* fiberoptic bronchoscopic, *TMP/SMX* trimethoprim-sulfamethoxazole, *VOR* voriconazole, *MEM* meropenem

Recent decades have witnessed the molecular understanding of the mechanisms involved in PH. PH1 is determined by mutations *of AGXT* gene (Located: 2q 37.3, contains 11 exons), which is wide phenotypic variability. Out of 253 known PVs in 358 variants of *AGXT* gene, 4 PVs of *AGXT* in PH1 patients (c.33dupC, c.454T>A, c.508G>A, and c.731T>C) were the most common in the reported PH1 mutations in the literature (http://www.ncbi.nlm.nih.gov/clinvar/). In the case 2, we detected the PV of *AGXT* gene (c.33dupC), which was last reviewed on Oct 9, 2015, and on top of that we first reported the novel mutation of *AGXT* gene (c.215A > T). Apart from this, the potential role of the missense mutation in *MUT* gene (c.1897G > C) in the development of the patient’s disease needed further confirmation.

The diagnosis of PH2 is established by the identification of PV in *GRHPR* (Located: 9p13.2, contains 11 exons). Out of 81 known PVs in 159 variants of *GRHPR* gene, 3 PVs in PH2 patients (c.103delG, c.295C>T, and c.864_865delTG) were the most common in the reported PH2 mutations in the literature (http://www.ncbi.nlm.nih.gov/clinvar/). In our case 1, we not only confirmed the PV (c.295C>T, which was last reviewed on Nov 2, 2017) inherited from his mother, but also detected the novel mutations (c.512G > A, c.211G > A) inherited from his father and passed them to his son. Although both the patient’s father and his son were the asymptomatic carrier without renal stones, they still needed closely follow-up observation.

The diagnostic findings of PH3 are PV in *HOGA1* (Located: 10q24.2, contains 7 exons). Out of 42 known PVs in 118 variants of *HOGA1* gene, 2 PVs in PH3 patients (c.700+5G>T and c.944_946delAGG) were the most common in the reported PH3 mutations in the literature (http://www.ncbi.nlm.nih.gov/clinvar/). PH3 has not been reported yet in our hospital till now.

In the light of the poor prognosis of the recurrent PH and the limitations of conservative measures (including overhydration, crystallization inhibitors and PN) as well as the risk of renal replacement therapy (despite an inevitable fact that shows the superiority of LKT over KAT) [17, 18], the confirmation of the genetic defect in PH patients had urged us to seek novel therapeutic approaches to control the disease without the need for LKT or KAT, and the emerging molecular therapy for PH may be feasible. The molecular therapy, such as enzyme replacement, substrate reduction, proteostasis regulation, as well as gene and cell therapy, may fix the mess that the overplus of oxalate is produced by mutant hepatocytes owing to the abnormal metabolism of glyoxylate in PH patients [19].

Despite the replacement enzyme need to be targeted to a specific subcellular organelle, the peroxisome, and the excessive oxalate would proceed to be formed in those untargeted hepatocytes, Roncador et al. [20] described the delivering AGT via the conjugation with PEG-PGA to the peroxisomes, as well as Mesa-Torres et al. [21] reported the use of pharmacological ligands aimed to increase AGT stability as therapies for PH1, which suggested enzyme replacement therapy (ERT) might play a role in PH.

Glycolate oxidase (GO) is the only member of the α-hydroxyacid oxidase family that transforms glycolate into glyoxylate [22]. Martin-Higueras et al. presented proof-of-concept evidence for substrate reduction therapy (SRT) in PH1 that GO inhibitors reduced the oxalate production by inhibiting GO activity [23]. Dutta et al. verified that inhibition of GO with Dicer-substrate siRNA had reduced CaOx deposition in a mouse model of PH1 [24]. Moreover, several ongoing clinical trials are currently exploring the safety and effectiveness of the therapy of the GO silencing in PH patients [19].

It’s well known that the administration of pyridoxine hydrochloride (vitamin B6) proposed as chaperone- proteostasis regulator therapy (CPRT) in PH1 several decades ago was ascribed to the fact that pyridoxal 5′-phosphate (PLP, a form of vitamin B6) is a cofactor for AGT. Even more, other B6 vitamers, such as pyridoxamine (PM) and pyridoxal (PL), were reported to be more effective than PN in rescuing folding-defective variants of human alanine in PH1 [25, 26].

Gene therapy in PH has been tested in several preclinical studies [27, 28]. Recently Castello et al. investigated Helper-dependent adenoviral vectors (HDAdV) for liver-directed gene therapy in the mouse PH1 model to achieve long-term liver transgene expression and correction of hyperoxaluria after a single injection [29].

Since KAT does not correct the underlying metabolic defect, just like the 3 cases we reported, transplant recipients have a high risk of recurrence of crystalline nephropathy which can lead to graft loss, as well as LKT is limited by the scarcity of organs and the need for long-term immunosuppression [30], Zapata-Linares et al. attempted the cell therapy by using hepatocytes generated from human induced pluripotent stem cells (hiPSCs) derived from a PH1 patient with p. I244T mutation as vehicles for ex vivo gene therapy using autologous cells to circumvent the immunosuppression [31].

In summary, our above case report indicated that diagnosed PH after kidney transplantation failure prompts us to execute preoperative screening of PH in all patients even with a minor history of nephrolithiasis and seek the better methods of treatments to avoid tragedies.

## Additional files

Additional file 1: **Figure S1.** The results of laboratory tests of case 1. The SCr and BUN of the patient were charted serially, showing the course of the delayed graft function (DGF), and accompanied by the occasional fever of 37.7 °C. Meanwhile, the levels of WBC count and neutrophils significantly fluctuated, and the Tac blood concentrations ranged from 2.6 to 19.3 ng/ml. (TIF 2161 kb)

Additional file 2: **Figure S2.** The results of laboratory tests of case 2. The SCr and BUN of the patient were charted serially, showing the development of DGF, even re-progression to ESRD. In the meantime, the levels of WBC count and neutrophils, as well as HGB fluctuated significantly. The Tac blood concentrations ranged from 5.6 to 9.0 ng/ml before replaced by Cyclosporine A (CsA), which hereafter ranged from 82.7 to 258.3 ng/ml. (TIF 2269 kb)

Additional file 3: **Figure S3.** The results of laboratory tests of case 3. The SCr and BUN of the patient were charted serially, showing the emergence of DGF and the renal transplant failure with fatal consequences, accompanied by the fever of 39.8 °C. Meanwhile, the levels of WBC count and neutrophils fluctuated significantly. The Tac blood concentrations ranged from 3.3 to 15.9 ng/ml before it was stopped. (TIF 2530 kb)

## Data Availability

The raw data that support the findings of this report are available from The Third Affiliated Hospital of Guangzhou Medical University and Guangzhou Kingmed Center for Clinical Laboratory Co., Ltd. but restrictions apply to the availability of these data, which were used under license for the current study, and so are not publicly available. Data are however available from the authors upon reasonable request and with permission of The Third Affiliated Hospital of Guangzhou Medical University and Guangzhou Kingmed Center for Clinical Laboratory Co., Ltd.

## Abbreviations

ATG: Thymoglobulin
BUN: Blood urea nitrogen
CaOx: Calcium oxalate
CRBC: Concentrated red blood cells
CsA: Cyclosporine A
d: day(s)
DGF: Delayed graft function
ESRD: End-stage renal disease
FOB: Fiberoptic bronchoscopic
H&E: Hematoxylin and eosin
HD: Haemodialysis
HGB: Hemoglobin
IV: Intravenously
KAT: Kidney-alone transplantation
LKT: combined Liver-Kidney Transplantation
MEM: Meropenem
Methylpred: Methylprednisolone
MMF: Mycophenolate mofetil
PH: Primary hyperoxaluria
PJP: *Pneumocystis jirovecii* pneumonia
post-op: post-operative
PV: Pathogenic variant
QOD: Every other day
rhEPO: recombinant human erythropoietin
SCr: Serum creatinine
Tac: Tacrolimus
TCMR: T cell-mediated rejection
TMP/SMX: Trimethoprim-sulfamethoxazole
USG: Ultrasonography
VOR: Voriconazole
VUS: Variant of uncertain significance
WBC: White blood cell(s)
ZHIB: Zero-Hour Implantation biopsy
