# Supplementary figures and images for: Primary hyperoxaluria diagnosed after kidney transplantation failure: lesson from 3 case reports and literature review
Source: BMC Nephrol. 2019 Jun 18;20:224. doi: 10.1186/s12882-019-1402-2 (PMC6582561; doi:10.1186/s12882-019-1402-2)

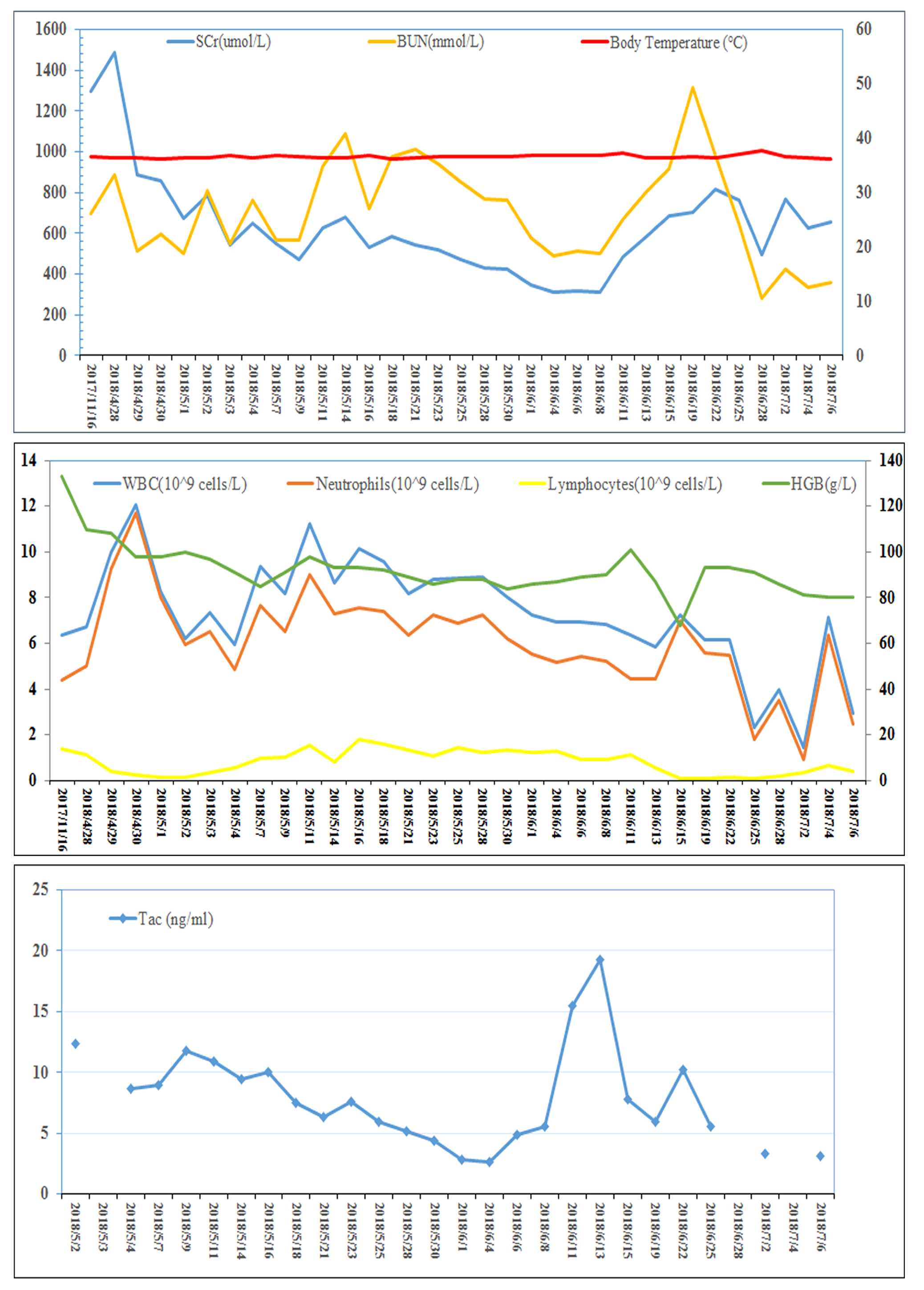

Supplement: Supplementary file 1 — Figure S1. The results of laboratory tests of case 1. The SCr and BUN of the patient were charted serially, showing the course of the delayed graft function (DGF), and accompanied by the occasional fever of 37.7 °C. Meanwhile, the levels of WBC count and neutrophils significantly fluctuated, and the Tac blood concentrations ranged from 2.6 to 19.3 ng/ml. (TIF 2161 kb) [file 12882_2019_1402_MOESM1_ESM.tif]

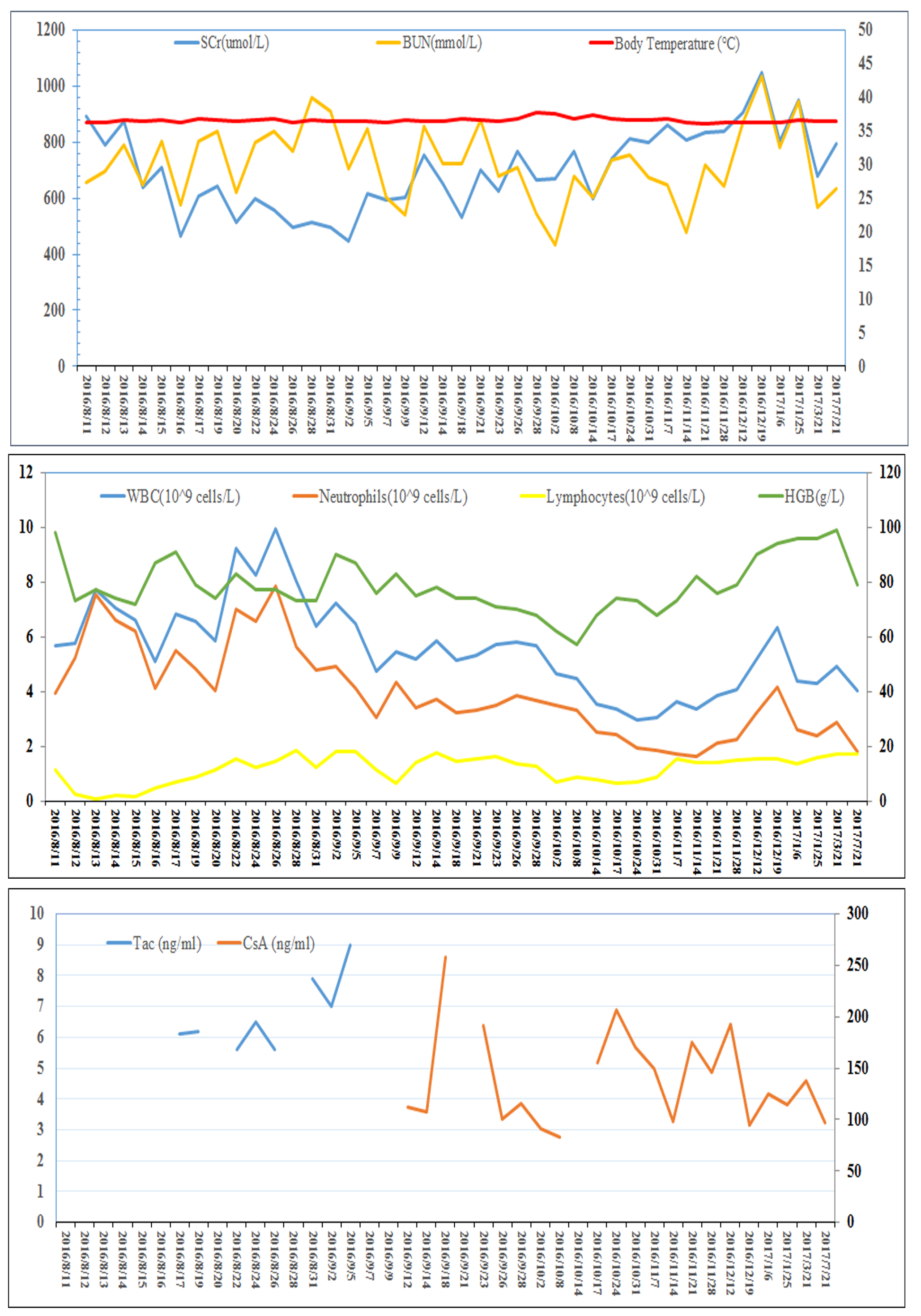

Supplement: Supplementary file 2 — Figure S2. The results of laboratory tests of case 2. The SCr and BUN of the patient were charted serially, showing the development of DGF, even re-progression to ESRD. In the meantime, the levels of WBC count and neutrophils, as well as HGB fluctuated significantly. The Tac blood concentrations ranged from 5.6 to 9.0 ng/ml before replaced by Cyclosporine A (CsA), which hereafter ranged from 82.7 to 258.3 ng/ml. (TIF 2269 kb) [file 12882_2019_1402_MOESM2_ESM.tif]

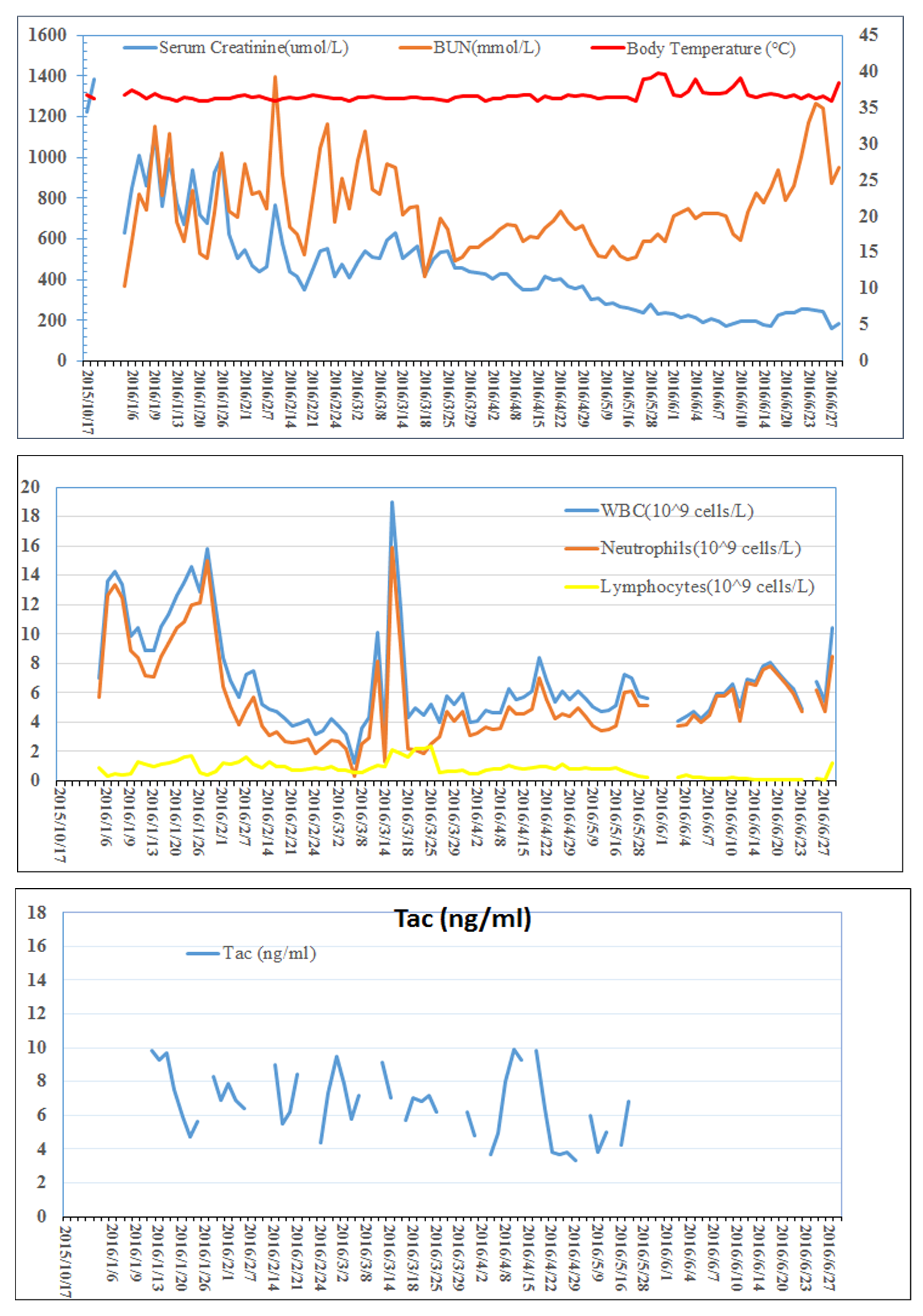

Supplement: Supplementary file 3 — Figure S3. The results of laboratory tests of case 3. The SCr and BUN of the patient were charted serially, showing the emergence of DGF and the renal transplant failure with fatal consequences, accompanied by the fever of 39.8 °C. Meanwhile, the levels of WBC count and neutrophils fluctuated significantly. The Tac blood concentrations ranged from 3.3 to 15.9 ng/ml before it was stopped. (TIF 2530 kb) [file 12882_2019_1402_MOESM3_ESM.tif]
